# Supplementary material for: Yield prediction for crops by gradient-based algorithms
Source: PLoS One. 2024 Aug 26;19(8):e0291928. doi: 10.1371/journal.pone.0291928 (PMC11346950; doi:10.1371/journal.pone.0291928)
Supplement: Attachment — Submitted filename: Response to Reviewers.docx [file pone.0291928.s001.docx]

**Manuscript Number:** PONE-D-23-17460

**Title:** **Yield Prediction for Crops by Gradient-Based Algorithms.**

Dear editors and reviewers,

Thank you for your letter and for the reviewers’ comments on our manuscript entitled “Yield Prediction for Crops by Gradient-Based Algorithms”. All of these comments were very helpful for revising and improving our paper. We have studied these comments carefully and have made corresponding corrections that we hope will meet with your approval. The changes made in the revised paper are summarized in our response below.

We would like to express our great appreciation to you and the reviewers for the comments on our paper. If you have any further queries, please do not hesitate to contact us.

Yours sincerely,

Pavithra M, Rajkumar S

- A rebuttal letter that responds to each point raised by the academic editor and reviewer(s). You should upload this letter as a separate file labeled 'Response to Reviewers.
- A marked-up copy of your manuscript that highlights changes made to the original version. You should upload this as a separate file labeled 'Revised Manuscript with Track Changes'.
- An unmarked version of your revised paper without tracked changes. You should upload this as a separate file labeled 'Manuscript'.

If you would like to make changes to your financial disclosure, please include your updated statement in your cover letter. Guidelines for resubmitting your figure files are available below the reviewer comments at the end of this letter.

1. Please ensure that your manuscript meets PLOS ONE's style requirements, including those for file naming. The PLOS ONE style templates can be found at 
<https://journals.plos.org/plosone/s/file?id=wjVg/PLOSOne_formatting_sample_main_body.pdf> and 
<https://journals.plos.org/plosone/s/file?id=ba62/PLOSOne_formatting_sample_title_authors_affiliations.pdf>

Author Response: We would like to thank the reviewer for pointing out this. We have revised the paper presented in PLOS ONE Format

2. Please note that PLOS ONE has specific guidelines on code sharing for submissions in which author-generated code underpins the findings in the manuscript. In these cases, all author-generated code must be made available without restrictions upon publication of the work. Please review our guidelines at <https://journals.plos.org/plosone/s/materials-and-software-sharing#loc-sharing-code> and ensure that your code is shared in a way that follows best practices and facilitates reproducibility and reuse.

Autor Response: Noted and the code will be shared after receiving acceptance for the article for publication

3. Thank you for stating the following financial disclosure: 
“The funders had no role in study design, data collection and analysis, decision to publish, or preparation of the manuscript.”

a) Please clarify the sources of funding (financial or material support) for your study. List the grants or organizations that supported your study, including funding received from your institution.

b) State what role the funders took in the study. If the funders had no role in your study, please state: “The funders had no role in study design, data collection, and analysis, decision to publish, or preparation of the manuscript.”
c) If any authors received a salary from any of your funders, please state which authors and which funders.

d) If you did not receive any funding for this study, please state: “The authors received no specific funding for this work.”

Autor Response: No funding has been received for the study. We have mentioned this in the paper as advised.

Please include your amended statements within your cover letter; we will change the online submission form on your behalf.

4. We note that you have stated that you will provide repository information for your data at acceptance. Should your manuscript be accepted for publication, we will hold it until you provide the relevant accession numbers or DOIs necessary to access your data. If you wish to make changes to your Data Availability statement, please describe these changes in your cover letter and we will update your Data Availability statement to reflect the information you provide.

Author Response: The dataset used for this research is publicly and freely available on the internet. We described data availability in the cover letter.

5. PLOS requires an ORCID iD for the corresponding author in Editorial Manager on papers submitted after December 6th, 2016. Please ensure that you have an ORCID iD and that it is validated in Editorial Manager. To do this, go to ‘Update my Information’ (in the upper left-hand corner of the main menu), and click on the Fetch/Validate link next to the ORCID field. This will take you to the ORCID site and allow you to create a new iD or authenticate a pre-existing iD in Editorial Manager. Please see the following video for instructions on linking an ORCID iD to your Editorial Manager account: <https://www.youtube.com/watch?v=_xcclfuvtxQ>

Autor Response: The corresponding author (Rajkumar S ) has an ORCID iD: 0000-0001-5701-9325

Additionally, please do the following:

-Visualization of obtained results must be improved.

Autor Response: We have improved visualization of obtained results in the revised manuscript.

- Motivation behind proposed research should be more clearly explain.

Author Response: We would like to thank the reviewer for raising this important point in the motivation of the study. We have enhanced the motivation of the study where it reads; [#page 4]

- Please elaborate what is "beyond state-of-the-art" of proposed. study.

Author Response: The main intent of the study is to verify the effectiveness of the gradient methods and the algorithms are finely modified to avoid overfitting and underfitting. The same is mentioned in the Introduction.

- To prove the significance of obtained results, statistical tests must be conducted.
- There are many statistical tests appropriate for validating results, please choose some tests from the following reference: <https://www.sciencedirect.com/science/article/pii/S2210650211000034>
  - For the sake of clarity, best obtained metrics in each table should be marked e.g. by using the bold style.
- Make sure that the source code is available according to PLOS ONE publication policies.
- [Note: HTML markup is below. Please do not edit.]

Author Response: We would like to thank the reviewer for raising this important point .As per reviewer comments we have conducted one way ANOVA statistical test to prove the significants of obtained results. [Page#21-22].

**Comments to the Author**

1. Is the manuscript technically sound, and do the data support the conclusions?

The manuscript must describe a technically sound piece of scientific research with data that supports the conclusions. Experiments must have been conducted rigorously, with appropriate controls, replication, and sample sizes. The conclusions must be drawn appropriately based on the data presented.

Reviewer #1: Yes

Reviewer #2: Partly

2. Has the statistical analysis been performed appropriately and rigorously?

Reviewer #1: Yes

Reviewer #2: No

3. Have the authors made all data underlying the findings in their manuscript fully available?

The [PLOS Data policy](http://www.plosone.org/static/policies.action#sharing) requires authors to make all data underlying the findings described in their manuscript fully available without restriction, with rare exception (please refer to the Data Availability Statement in the manuscript PDF file). The data should be provided as part of the manuscript or its supporting information, or deposited to a public repository. For example, in addition to summary statistics, the data points behind means, medians and variance measures should be available. If there are restrictions on publicly sharing data—e.g. participant privacy or use of data from a third party—those must be specified.

Reviewer #1: Yes

Reviewer #2: Yes

4. Is the manuscript presented in an intelligible fashion and written in standard English?

PLOS ONE does not copyedit accepted manuscripts, so the language in submitted articles must be clear, correct, and unambiguous. Any typographical or grammatical errors should be corrected at revision, so please note any specific errors here.

Reviewer #1: No

Reviewer #2: Yes

5. Review Comments to the Author

Reviewer #1: This manuscript evaluates various machine learning algorithms used to predict product performance. The authors compare the results of three machine learning algorithms, CatBoost, LightGBM and XGBoost. Their results show that CatBoost has the highest accuracy in yield prediction.
I went through the manuscript carefully. At the detailed level, the following notes are my suggestions:
1) Although the ABSTRACT structure is good, I suggest removing the first 5-6 lines and the last 3-4 lines. They are not really the descriptions that the reader expects to see in the ABSTRACT.

Author Response: We would like to thank the reviewer for raising this important point in the abstract of the study. We have enhanced the abstract of the study where it reads;

Abstract: A timely and consistent assessment of crop yield will assist the farmers in improving their income, minimizing losses, and deriving strategic plans in agricultural commodities to adopt import-export policies. Crop yield predictions are one of the various challenges faced in the agriculture sector and play a significant role in planning and decision-making. Machine learning algorithms provided enough belief and proved their ability to predict crop yield. The selection of the most suitable crop is influenced by various environmental factors such as temperature, soil fertility, water availability, quality, and seasonal variations, as well as economic considerations such as stock availability, preservation capabilities, market demand, purchasing power, and crop prices. The paper outlines a framework used to evaluate the performance of various machine-learning algorithms for forecasting crop yields. The models were based on a range of prime parameters including pesticides, rainfall & avg. temperature. The Results of three machine learning algorithms, Categorical Boosting (CatBoost), Light Gradient-Boosting Machine LightGBM, and Extreme Gradient Boosting XGBoost are compared and found more accurate than other algorithms in predicting crop yields. The RMSE and R2 values were calculated to compare the predicted and observed rice yields, resulting in the following values: Cat-Boost with 800 (0.24), LightGBM with 737 (0.33), and XGBoost with 744 (0.31). Among these three machine learning algorithms, CatBoost demonstrated the highest precision in predicting yields, achieving an accuracy rate of 99.123%.

2) In my opinion, the INTRODUCTION section needs to be revised. In this section there should be three points: 1) motivation, 2) a summary of the challenges of previous studies, and 3) contribution. Also, the research contributions should be mentioned in a bullet-form at the end of the INTRODUCTION.

Author Response: We would like to thank the reviewer for raising this important point in the motivation of the study. We have enhanced the motivation of the study where it reads; [Page#4]

Motivation of the study is to develops an effective machine learning-based approach to crop yield prediction. Model a Prediction tool based on accurate crop yield predictions that can assist farmers and decision-makers about crop management, resource allocation, and risk mitigation strategies. With the uncertain weather patterns due to global warming, such a tool can be particularly useful in helping farmers adapt to changing conditions and ensure food security for the growing population.

Author Response: Summary of the challenges of previous studies has been included in the revised manuscript [Page#3]

Author Response: Contribution of the research have been mentioned in bullet-form at the end of the Introduction [Page#5]

Author Response: The main contribution of the work presented in this paper is outlined below

- Leveraging publicly available data on weather, agricultural practices, pesticides, and chemicals, a predictive model capable of accurately forecasting crop yields in India have been developed .
- One-Hot Encoding has been used to convert categorical variables to the one-hot numeric array
- Three different machine learning algorithms (CatBoost, LightGBM, and XGBoost) has been adopted in the model for achieving accurate prediction results for crops
- The developed robust prediction framework has been modelled to effectively avoid overfitting and underfitting scenarios.

3) The INTRODUCTION section is too long. It is almost 10 pages! Instead, authors should reduce the INTRODUCTION to 2 pages. Then add a section called RELATED WORKS that provides a summary of previous studies. Also having subsection 1-1 doesn't help. Merge it with Section 1.

Author Response: We would like to thank the reviewer for raising this important point in the introduction of the study. We have to reduce the introduction section in two pages [Page#2]

Author Response: We have included the following articles in the related works [Page#5].

[9]Jeong JH, Resop JP, Mueller ND, Fleisher DH, Yun K, et al. (2016) Random Forests for Global and Regional Crop Yield Predictions. PLOS ONE 11(6): e0156571. <https://doi.org/10.1371/journal.pone.0156571>

[13]Jovanovic, G.; Perisic, M.; Bacanin, N.; Zivkovic, M.; Stanisic, S.; Strumberger, I.; Alimpic, F.; Stojic, A. Potential of Coupling Metaheuristics-Optimized-XGBoost and SHAP in Revealing PAHs Environmental Fate. Toxics 2023, 11, 394. <https://doi.org/10.3390/toxics11040394>

[14] Jui-Sheng Chou, Chi-Yun Liu, Handy Prayogo, Riqi Radian Khasani, Danny Gho, Gretel Gaby Lalitan, Predicting nominal shear capacity of reinforced concrete wall in building by metaheuristics-optimized machine learning, Journal of Building Engineering, Volume 61, 2022, https://doi.org/10.1016/j.jobe.2022.105046.

[15] Zivkovic M, Tair M, K V, Bacanin N, Hubálovský Š, Trojovský P. 2022. Novel hybrid firefly algorithm: an application to enhance XGBoost tuning for intrusion detection classification. PeerJ Computer Science 8:e956 <https://doi.org/10.7717/peerj-cs.956>

[16] A. Petrovic, I. Strumberger, M. Antonijevic, D. Jovanovic, D. Mladenovic and A. Chabbra, "Firefly-Xgboost Approach for Pedestrian Detection," 2022 IEEE Zooming Yield Prediction for Crops by Gradient Based Algorithms 19 / 19 Innovation in Consumer Technologies Conference (ZINC), Novi Sad, Serbia, 2022, pp. 197- 202, doi: 10.1109/ZINC55034.2022.9840700.

[17] Jovanovic, L. et al. (2023). Tuning XGBoost by Planet Optimization Algorithm: An Application for Diabetes Classification. In: Bindhu, V., Tavares, J.M.R.S., Vuppalapati, C. (eds) Proceedings of Fourth International Conference on Communication, Computing and Electronics Systems. Lecture Notes in Electrical Engineering, vol 977. Springer, Singapore. https://doi.org/10.1007/978-981-19-7753-4_60

[18] Petrovic, A., Antonijevic, M., Strumberger, I., Jovanovic, L., Savanovic, N., & Janicijevic, S. (2023, January). The XGBoost Approach Tuned by TLB Metaheuristics for Fraud Detection. In Proceedings of the 1st International Conference on Innovation in Information Technology and Business (ICIITB 2022) (Vol. 104, p. 219). Springer Nature.

[23] Shook J, Gangopadhyay T, Wu L, Ganapathysubramanian B, Sarkar S, et al. (2021) Crop yield prediction integrating genotype and weather variables using deep learning. PLOS ONE 16(6): e0252402. <https://doi.org/10.1371/journal.pone.0252402>

4) It is not clear to me which formulas were invented by the authors themselves and which ones are derived from other references. I found evidence that some formulas are derived from other references and there are similarities.

Author Response: The formulae are adopted from reference papers.[not invented by authors] The basic equations are similar/same and will not change. However we have framed objective function from the basic equations to suit crop prediction.

5) Authors should also use common supervised learning metrics such as accuracy, precision, and recall. For this purpose, I recommend adding the following reference and using the definitions of the above metrics from there:
<https://www.tandfonline.com/doi/abs/10.1080/0952813X.2022.2153279>

Author Response: We agree with the reviewer, the metric mentioned are commonly used. But We have adopted the metrics used in reference paper to compare the results. Hence kindly excuse for not adding the reference recommended.

6) There are still some grammatical errors in the manuscript. Authors should use software such as Grammarly for proof-checking.

Author Response: Thank you for pointing grammatical errors in our manuscript. We have carefully studied the manuscript and minimized the grammatical mistakes using Grammarly.

7) The tense of the verbs in the CONCLUSION section must be past tense. In this section, the most important numerical improvements of the proposed method should be mentioned and marginal explanations should be avoided. Also, the suggestions mentioned for further research should be presented in a new paragraph.

Author Response: We would like to thank the reviewer for raising this important point in the conclusion of the study. We have enhanced the conclusion and add future scope of the study where it reads; [#page 17]

**Reviewer #2:**

1. Try to avoid using acronyms in the abstract.

Author Response: Thank you for pointing this comment. We shortened the briefing acronyms in the abstract.

2. Introduction is too long, please reduce it to max two pages.

Author Response: We would like to thank the reviewer for raising this important point in the introduction of the study. We have to reduce the introduction section in two pages[Page#2]

3. Provide additional section - Background and related work, where you should provide literature survey.

Author Response: Author Response: We have included the following articles in the related works [Page#5].

4. Literature background must be expanded, as it is very limited. Include more recent relevant papers dealing with metaheuristics-based models, to provide stronger background on ML models. Include the following:
<https://www.mdpi.com/2305-6304/11/4/394>
<https://www.sciencedirect.com/science/article/abs/pii/S2352710222010555>
<https://peerj.com/articles/cs-956/>
<https://ieeexplore.ieee.org/abstract/document/9840700>
<https://link.springer.com/chapter/10.1007/978-981-19-7753-4_60>
<https://www.atlantis-press.com/proceedings/iciitb-22/125984192>

Author Response: As per the reviewer’s suggestion we expanded the literature background by including the following most recent metaheuristics-based and ML models in the revised manuscript.

[9]Jeong JH, Resop JP, Mueller ND, Fleisher DH, Yun K, et al. (2016) Random Forests for Global and Regional Crop Yield Predictions. PLOS ONE 11(6): e0156571. <https://doi.org/10.1371/journal.pone.0156571>

[13]Jovanovic, G.; Perisic, M.; Bacanin, N.; Zivkovic, M.; Stanisic, S.; Strumberger, I.; Alimpic, F.; Stojic, A. Potential of Coupling Metaheuristics-Optimized-XGBoost and SHAP in Revealing PAHs Environmental Fate. Toxics 2023, 11, 394. <https://doi.org/10.3390/toxics11040394>

[14] Jui-Sheng Chou, Chi-Yun Liu, Handy Prayogo, Riqi Radian Khasani, Danny Gho, Gretel Gaby Lalitan, Predicting nominal shear capacity of reinforced concrete wall in building by metaheuristics-optimized machine learning, Journal of Building Engineering, Volume 61, 2022, https://doi.org/10.1016/j.jobe.2022.105046.

[15] Zivkovic M, Tair M, K V, Bacanin N, Hubálovský Š, Trojovský P. 2022. Novel hybrid firefly algorithm: an application to enhance XGBoost tuning for intrusion detection classification. PeerJ Computer Science 8:e956 <https://doi.org/10.7717/peerj-cs.956>

[16] A. Petrovic, I. Strumberger, M. Antonijevic, D. Jovanovic, D. Mladenovic and A. Chabbra, "Firefly-Xgboost Approach for Pedestrian Detection," 2022 IEEE Zooming Yield Prediction for Crops by Gradient Based Algorithms 19 / 19 Innovation in Consumer Technologies Conference (ZINC), Novi Sad, Serbia, 2022, pp. 197- 202, doi: 10.1109/ZINC55034.2022.9840700.

[17] Jovanovic, L. et al. (2023). Tuning XGBoost by Planet Optimization Algorithm: An Application for Diabetes Classification. In: Bindhu, V., Tavares, J.M.R.S., Vuppalapati, C. (eds) Proceedings of Fourth International Conference on Communication, Computing and Electronics Systems. Lecture Notes in Electrical Engineering, vol 977. Springer, Singapore. https://doi.org/10.1007/978-981-19-7753-4_60

[18] Petrovic, A., Antonijevic, M., Strumberger, I., Jovanovic, L., Savanovic, N., & Janicijevic, S. (2023, January). The XGBoost Approach Tuned by TLB Metaheuristics for Fraud Detection. In Proceedings of the 1st International Conference on Innovation in Information Technology and Business (ICIITB 2022) (Vol. 104, p. 219). Springer Nature

5. Figures should be provided in much better quality.

Author Response: Thank you for raising this important point. We have presented figures with better legibility.

6. Elaborate in more details why gradient boosting methods were selected for this task, among other ML models.

Author Response: The selected ML Methods has the potential to fit the crop prediction problem and have not been implemented for similar work. Reasons briefed in the introduction part

7. The proposed model should be explained in more details.

Author Response: We believe, the model has been briefed in the paper.

8. There are some errors in equations (square signs where operators should be).

Author Response : Thank you for pointing out errors in equations. We have carefully studied and corrected those errors in the revised manuscript.

9. Make sure that each parameter in every equation has been explained in the text.

Author Response: Noted, the significant parameters have been explained in the manuscript.

10. Elaborate in more details how the hyperparameters for the models were selected.

Author Response: Distributed grid search method, utilizing GridSearchCV from the Python sci-kit-learn library. The same has been mentioned in the paper.

11. Finally, I would recommend performing a statistical analysis, to establish if the obtained results are statistically significant in comparison to other methods.

Author Response: We completely agree with the reviewer’s recommendation to include statistical analysis. The inclusion of such analysis will improve the content of the study. As per reviewer comments we have conducted one way ANOVA statistical test to prove the significants of obtained results. [Page#21-22].

12. Paper needs extensive proofreading, as there are numerous grammatical and language errors in the text.

Author Response: Thank you for pointing out grammatical and language errors in our manuscript. We have carefully studied the manuscript and minimized the grammatical mistakes using Grammarly.

6. PLOS authors have the option to publish the peer review history of their article ([what does this mean?](https://journals.plos.org/plosone/s/editorial-and-peer-review-process#loc-peer-review-history)). If published, this will include your full peer review and any attached files.

If you choose “no”, your identity will remain anonymous but your review may still be made public.

**Do you want your identity to be public for this peer review?** For information about this choice, including consent withdrawal, please see our [Privacy Policy](https://www.plos.org/privacy-policy).

Reviewer #1: No

Reviewer #2: No
