# Supplementary material for: Yield prediction for crops by gradient-based algorithms
Source: PLoS One. 2024 Aug 26;19(8):e0291928. doi: 10.1371/journal.pone.0291928 (PMC11346950; doi:10.1371/journal.pone.0291928)
Supplement: Attachment — Submitted filename: Response to Reviewers.docx [file pone.0291928.s002.docx]

**Response to Reviewers**

Manuscript Number: PONE-D-23-17460R1

Title: Yield Prediction for Crops by Gradient-Based Algorithms

PLOS ONE

Dear editors and reviewers,

Thank you for consideration and for your efforts to bring the work in better way. We hereby submit a revised version of the manuscript that addresses the points raised during the review process.

Please find below our response to each point raised by the academic editor and reviewer(s).

We here by submit the below documents along with response to reviewers:

- A separate file labelled 'Revised Manuscript with Track Changes' is uploaded. The marked-up copy of your manuscript that highlights changes made to the original version.
- A separate file labelled 'Manuscript' An unmarked version of the revised paper without tracked changes.

1. If the authors have adequately addressed your comments raised in a previous round of review and you feel that this manuscript is now acceptable for publication, you may indicate that here to bypass the “Comments to the Author” section, enter your conflict-of-interest statement in the “Confidential to Editor” section, and submit your "Accept" recommendation.

Reviewer #1: All comments have been addressed

Reviewer #2: All comments have been addressed

Reviewer #3: (No Response)

Reviewer #4: (No Response)

Reviewer #5: All comments have been addressed

**Author Response:** No response required for the comment, since reviewers have concurred for addressing the comments

2. Is the manuscript technically sound, and do the data support the conclusions?

The manuscript must describe a technically sound piece of scientific research with data that supports the conclusions. Experiments must have been conducted rigorously, with appropriate controls, replication, and sample sizes. The conclusions must be drawn appropriately based on the data presented.

 Reviewer #1: Yes

Reviewer #2: (No Response)

Reviewer #3: No

Reviewer #4: Yes

Reviewer #5: Yes

**Author Response:** Performance Evaluation of Catboost, XGBoost, and LightGBM by Diebold-Mariano test for comparison has been additionally included and except one reviewer, other feel the results presented in the paper is adequate.

 3. Has the statistical analysis been performed appropriately and rigorously?

 Reviewer #1: Yes

Reviewer #2: (No Response)

Reviewer #3: No

Reviewer #4: Yes

Reviewer #5: Yes

**Author Response:** Statistical analysis by Diebold-Mariano test has been additionally included and except one reviewer, other feel the results presented in the paper is adequate.

 4. Have the authors made all data underlying the findings in their manuscript fully available?

The [PLOS Data policy](https://ind01.safelinks.protection.outlook.com/?url=http%3A%2F%2Fwww.plosone.org%2Fstatic%2Fpolicies.action%23sharing&data=05%7C01%7Cramesh.r%40epd.bgrenergy.com%7Cb30ee02af74349f6963408dbf289d927%7Ce5fa0c2494c1414d93f6592acd5489b8%7C1%7C0%7C638370446315042936%7CUnknown%7CTWFpbGZsb3d8eyJWIjoiMC4wLjAwMDAiLCJQIjoiV2luMzIiLCJBTiI6Ik1haWwiLCJXVCI6Mn0%3D%7C1000%7C%7C%7C&sdata=ducgbItjatsLQ1oJBgXGzwPzyURmxx2AT0hBRbZSxao%3D&reserved=0) requires authors to make all data underlying the findings described in their manuscript fully available without restriction, with rare exception (please refer to the Data Availability Statement in the manuscript PDF file). The data should be provided as part of the manuscript or its supporting information, or deposited to a public repository. For example, in addition to summary statistics, the data points behind means, medians and variance measures should be available. If there are restrictions on publicly sharing data—e.g. participant privacy or use of data from a third party—those must be specified.

 Reviewer #1: No

Reviewer #2: (No Response)

Reviewer #3: No

Reviewer #4: Yes

Reviewer #5: No

**Author Response:** Dataset obtained from third party used for experimentation has been referred in the paper [24] and results obtained during experimentation have been recorded fully in the paper. If information in specific is required, kindly mention. The authors are willing to send.

 5. Is the manuscript presented in an intelligible fashion and written in standard English?

PLOS ONE does not copyedit accepted manuscripts, so the language in submitted articles must be clear, correct, and unambiguous. Any typographical or grammatical errors should be corrected at revision, so please note any specific errors here.

 Reviewer #1: Yes

Reviewer #2: (No Response)

Reviewer #3: No

Reviewer #4: Yes

Reviewer #5: Yes

**Author Response:** It can be noted the reviewers (except Reviewer #3) considered the paper is written in standard English and in better manner. Considering the Reviewer #3 response, we have made few corrections for better understanding and reading.

6. Review Comments to the Author

Please use the space provided to explain your answers to the questions above. You may also include additional comments for the author, including concerns about dual publication, research ethics, or publication ethics. (Please upload your review as an attachment if it exceeds 20,000 characters)

 Reviewer #1: The authors appreciated my comments. I have no other comment. From my husband, this manuscript can be published.

Reviewer #2: (No Response)

Reviewer #3: The article presents a study on Yield Prediction for Crops by Gradient Based Algorithms. In comparison of various other studies published on crop prediction in other journals, I found that this article lacks novelty in terms of originality and technicality. The methods used for crop analysis is very trivial and outdated. Moreover, the results do not provide any useful information for agriculture domain as well. I would suggest a rejection.

**Author Response:** Kindly note, our intent is to investigate the capability of gradient methods for crop predictions. The experimentation results provide the proof for capacity of gradient methods. The results give us confident to enhance the model considering below aspects to reach a complete frame work.

- Integrated Framework Development
- Incorporation of Additional Environmental Factors
- Enhanced Predictive Models
- Continuous Environmental Monitoring System
- Exploration of Multi-Crop Recommendations
- Integration with Precision Agriculture Technologies
- Community Engagement and Farmer Education
- Long-Term Impact Assessment

Reviewer #4: 1. Introduction may be improved, adding the highlights and the problem statements.

**Author Response:** We would like to thank the reviewer for pointing out this. Problem statement with highlights of the work included in introduction of the revised paper [Page#3].

2. You could improve writing, link better the ideas flow in the Introduction.

**Author Response:** We would like to thank the reviewer for raising this important point in the introduction, the paper starts with aspects of framing, problem statement (now included), continued with a brief about IOT and data Analysis, motivation of the study, contributions and content covered in sessions of the paper. A paragraph of details to link the flow of ideas has been included.

3. Review references because some of them are unstandardized.

**Author Response:** We would like to thank the reviewer for raising this important point. As per reviewer comments we have included references from standard journals and revised the paper [Page#28-31].

4. The conclusion needs improvements towards major claimed contribution.

**Author Response:** As per the reviewer’s suggestion we have improved the conclusion toward major claimed contribution. [Page#26-27]

5. Write some future directions in the conclusion section.

**Author Response:** As per the suggestion of the reviewer the future directions have been included in conclusion section. [Page#26-27]

Reviewer #5: The comparative assessment of different models may be statistically tested. Authors may use Diebold Mariano test for this purpose. you may see following paper [https://doi.org/10.1371/journal](https://ind01.safelinks.protection.outlook.com/?url=https%3A%2F%2Fdoi.org%2F10.1371%2Fjournal&data=05%7C01%7Cramesh.r%40epd.bgrenergy.com%7Cb30ee02af74349f6963408dbf289d927%7Ce5fa0c2494c1414d93f6592acd5489b8%7C1%7C0%7C638370446315042936%7CUnknown%7CTWFpbGZsb3d8eyJWIjoiMC4wLjAwMDAiLCJQIjoiV2luMzIiLCJBTiI6Ik1haWwiLCJXVCI6Mn0%3D%7C1000%7C%7C%7C&sdata=R7L8cwvnb73qWfMrVj%2BbwS441wI7OP1BLC%2Bd1Bl3U8A%3D&reserved=0).
pone.0270553

MAPE (mean absolute prediction error) may also be reported.

**Author Response:** Thank you for raising this important comment. The comparative assessment of different models has been statistically tested by Diebold-Mariano test in revised paper [Page# 21-22]. Moreover, as per reviewer’s suggestion, we have included mean absolute percentage error [Page#19-20].

 7. PLOS authors have the option to publish the peer review history of their article ([what does this mean?](https://ind01.safelinks.protection.outlook.com/?url=https%3A%2F%2Fjournals.plos.org%2Fplosone%2Fs%2Feditorial-and-peer-review-process%23loc-peer-review-history&data=05%7C01%7Cramesh.r%40epd.bgrenergy.com%7Cb30ee02af74349f6963408dbf289d927%7Ce5fa0c2494c1414d93f6592acd5489b8%7C1%7C0%7C638370446315042936%7CUnknown%7CTWFpbGZsb3d8eyJWIjoiMC4wLjAwMDAiLCJQIjoiV2luMzIiLCJBTiI6Ik1haWwiLCJXVCI6Mn0%3D%7C1000%7C%7C%7C&sdata=2Erb0yIauIQrYNevHfU0zmu1dQL%2BEzmUA43DxNqswXw%3D&reserved=0)). If published, this will include your full peer review and any attached files.

If you choose “no”, your identity will remain anonymous but your review may still be made public.

**Do you want your identity to be public for this peer review?** For information about this choice, including consent withdrawal, please see our [Privacy Policy](https://ind01.safelinks.protection.outlook.com/?url=https%3A%2F%2Fwww.plos.org%2Fprivacy-policy&data=05%7C01%7Cramesh.r%40epd.bgrenergy.com%7Cb30ee02af74349f6963408dbf289d927%7Ce5fa0c2494c1414d93f6592acd5489b8%7C1%7C0%7C638370446315042936%7CUnknown%7CTWFpbGZsb3d8eyJWIjoiMC4wLjAwMDAiLCJQIjoiV2luMzIiLCJBTiI6Ik1haWwiLCJXVCI6Mn0%3D%7C1000%7C%7C%7C&sdata=Fo0auY%2FETgdppKP6W1B4MGDqtQgfKX2PNnmljDYeLd0%3D&reserved=0).

 Reviewer #1: No

Reviewer #2: No

Reviewer #3: No

Reviewer #4: No

Reviewer #5: No

**Author Response:** Noted

[NOTE: If reviewer comments were submitted as an attachment file, they will be attached to this email and accessible via the submission site. Please log into your account, locate the manuscript record, and check for the action link "View Attachments". If this link does not appear, there are no attachment files.]

While revising your submission, please upload your figure files to the Preflight Analysis and Conversion Engine (PACE) digital diagnostic tool, [https://pacev2.apexcovantage.com/](https://ind01.safelinks.protection.outlook.com/?url=https%3A%2F%2Fpacev2.apexcovantage.com%2F&data=05%7C01%7Cramesh.r%40epd.bgrenergy.com%7Cb30ee02af74349f6963408dbf289d927%7Ce5fa0c2494c1414d93f6592acd5489b8%7C1%7C0%7C638370446315042936%7CUnknown%7CTWFpbGZsb3d8eyJWIjoiMC4wLjAwMDAiLCJQIjoiV2luMzIiLCJBTiI6Ik1haWwiLCJXVCI6Mn0%3D%7C1000%7C%7C%7C&sdata=h4QKecZeaCget3bfpHoDwawnP0uRV21dtr66RHLvdH0%3D&reserved=0). PACE helps ensure that figures meet PLOS requirements. To use PACE, you must first register as a user. Registration is free. Then, login and navigate to the UPLOAD tab, where you will find detailed instructions on how to use the tool. If you encounter any issues or have any questions when using PACE, please email PLOS at [figures@plos.org](mailto:figures@plos.org). Please note that Supporting Information files do not need this step.

*In compliance with data protection regulations, you may request that we remove your personal registration details at any time.* [*(Remove my information/details)*](https://ind01.safelinks.protection.outlook.com/?url=https%3A%2F%2Fwww.editorialmanager.com%2Fpone%2Flogin.asp%3Fa%3Dr&data=05%7C01%7Cramesh.r%40epd.bgrenergy.com%7Cb30ee02af74349f6963408dbf289d927%7Ce5fa0c2494c1414d93f6592acd5489b8%7C1%7C0%7C638370446315199147%7CUnknown%7CTWFpbGZsb3d8eyJWIjoiMC4wLjAwMDAiLCJQIjoiV2luMzIiLCJBTiI6Ik1haWwiLCJXVCI6Mn0%3D%7C1000%7C%7C%7C&sdata=zvgQHImx%2FDeP%2FuQRzUxrvpDyTK%2BdfCL4uO1jQFFzQw4%3D&reserved=0)*. Please contact the publication office if you have any questions.*

***Disclaimer:*** *This message was sent from Vellore Institute of Technology.  The contents of this email may contain legally protected confidential or privileged information of “Vellore Institute of Technology”.  If you are not the intended recipient, you should not disseminate, distribute or copy this e-mail. Please notify the sender immediately and destroy all copies of this message and any attachments. If you have received this email in error, please promptly notify the sender by reply email and delete the original email and any backup copies without reading them.*

***Disclaimer:*** *This message was sent from Vellore Institute of Technology.  The contents of this email may contain legally protected confidential or privileged information of “Vellore Institute of Technology”.  If you are not the intended recipient, you should not disseminate, distribute or copy this e-mail. Please notify the sender immediately and destroy all copies of this message and any attachments. If you have received this email in error, please promptly notify the sender by reply email and delete the original email and any backup copies without reading them.*

Reply

Forward
